# 1,2,3,6,7,8-Hexahydro­cinnolino[5,4,3-*cde*]cinnoline

**DOI:** 10.1107/S1600536808044036

**Published:** 2009-01-08

**Authors:** Zhi-Qiang Gao

**Affiliations:** a College of Chemistry and Environmental Engineering, Chongqing University of Arts and Sciences, Chongqing, People’s Republic of China

## Abstract

The title compound, C_12_H_12_N_4_, was synthesized by the reaction of hydrazine hydrate  and 9-methyl-3,4,6,7-tetra­hydro-2*H*-xanthene-1,8(5*H*,9*H*)-dione in ethanol. In the crystal, the mol­ecule lies across an inversion centre. The pyridazine rings are coplanar and the C_6_ rings adopt envelope conformations.

## Related literature

For the biological properties of cinnoline and its derivatives, see: Abdelrazek *et al.* (2006 ▶); Gomtsyan *et al.* (2005 ▶); Inoue *et al.* (1993 ▶); Lewgowd & Stanczak (2007 ▶); Lewgowd *et al.* (2005 ▶); Singh *et al.* (2003 ▶); Stefanska *et al.* (2003 ▶); Tutsumi *et al.* (1992 ▶).
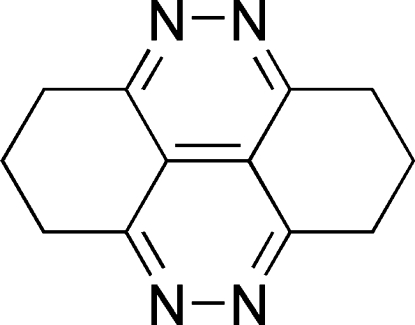


## Experimental

### 

#### Crystal data

C_12_H_12_N_4_
*M* *_r_* = 212.26
Monoclinic, 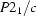

*a* = 9.698 (5) Å
*b* = 5.875 (3) Å
*c* = 10.023 (5) Å
β = 117.314 (6)°
*V* = 507.4 (4) Å^3^
*Z* = 2
Mo *K*α radiation
μ = 0.09 mm^−1^
*T* = 298 (2) K
0.55 × 0.41 × 0.09 mm

#### Data collection

Bruker SMART CCD area-detector diffractometer
Absorption correction: multi-scan (*SADABS*; Sheldrick, 1996 ▶) *T*_min_ = 0.953, *T*_max_ = 0.992
2508 measured reflections
890 independent reflections
575 reflections with *I* > 2σ(*I*)
*R*_int_ = 0.028

#### Refinement

*R*[*F* ^2^ > 2σ(*F* ^2^)] = 0.041
*wR*(*F* ^2^) = 0.123
*S* = 1.01
890 reflections
97 parameters
All H-atom parameters refined
Δρ_max_ = 0.16 e Å^−3^
Δρ_min_ = −0.15 e Å^−3^

### 

Data collection: *SMART* (Bruker, 1998 ▶); cell refinement: *SAINT* (Bruker, 1999 ▶); data reduction: *SAINT*; program(s) used to solve structure: *SHELXS97* (Sheldrick, 2008 ▶); program(s) used to refine structure: *SHELXL97* (Sheldrick, 2008 ▶); molecular graphics: *SHELXTL* (Sheldrick, 2008 ▶); software used to prepare material for publication: *SHELXTL*.

## Supplementary Material

Crystal structure: contains datablocks global, I. DOI: 10.1107/S1600536808044036/ci2750sup1.cif

Structure factors: contains datablocks I. DOI: 10.1107/S1600536808044036/ci2750Isup2.hkl

Additional supplementary materials:  crystallographic information; 3D view; checkCIF report

## Figures and Tables

**Fig. 1.**
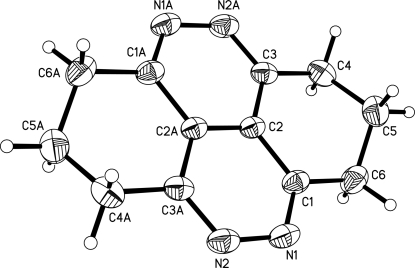
The molecular structure of the title compound, showing 50% probability displacement ellipsoids and the atom-numbering scheme. Atoms labelled with the suffix A are generated by the symmetry operation (1-x, -y, 1-z).

**Fig. 2.**
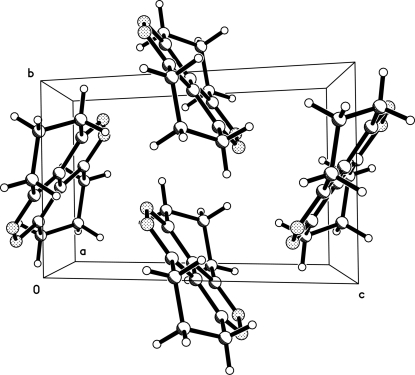
Molecular packing in the title compound, viewed approximately along the a axis.

## supplementary crystallographic information

### Comment

It is well known that six-membered nitrogen-containing heterocycles are abundant in numerous natural products that exhibit important biological properties. For example, cinnolines and their derivatives exhibit a diverse range of biological properties (Lewgowd & Stanczak, 2007) such as molluscicidal activity (Abdelrazek *et al.*, 2006), cytotoxic activity (Lewgowd *et al.*, 2005), transient receptor potential vanilloid 1 (TRPV1) receptor antagonists (Gomtsyan *et al.*, 2005), and topoisomerase I (TOP1)-targeting activity and cytotoxicity (Singh *et al.*, 2003). They also acted as anticancer agents active on a multidrug resistant cell line (Stefanska *et al.*, 2003). They can also be used as bactericides in pharmaceutical industry (Inoue *et al.*, 1993; Tutsumi *et al.*,1992). The chemistry of cinnolines has received much attention based on the above facts.

The title molecule lies across an inversion centre (Fig. 1). The two pyridazine rings (C1/C2/C2A/C3A/N2/N1 and C1A/C2A/C2/C3/N2A/N1A) are conjugated and are coplanar. The two cyclohexene rings (C1—C6 and C1A—C6A) adopt envelope conformations, with atoms C5 and C5A deviate from the C1/C2/C3/C4/C6 and C1A/C2A/C3A/C4A/C6A planes by 0.656 (3) and 0.656 (3) Å, respectively.

A view of the crystal packing is shown in Fig.2.

### Experimental

The title compound was prepared by the reaction of 3,4,6,7-tetrahydro -9-methyl-2*H*-xanthene-1,8(5*H*,9H)-dione (2 mmol) and hydrazine hydrate 80% (8 mmol) in ethanol (8 ml) stirring at 353 K (yield 84%; m.p. 543–544 K). Single crystals suitable for X-ray diffraction were obtained from an ethanol solution by slow evaporation.

### Refinement

All H atoms were located in a difference Fourier map and refined freely [C-H = 0.95 (2)-1.04 (2) Å].

### Crystal data

**Table:**

C_12_H_12_N_4_	*F*(000) = 224
*M**_r_* = 212.26	*D*_x_ = 1.389 Mg m^−^^3^
Monoclinic, *P*2_1_/*c*	Melting point = 543–544 K
Hall symbol: -P 2ybc	Mo *K*α radiation, λ = 0.71073 Å
*a* = 9.698 (5) Å	Cell parameters from 734 reflections
*b* = 5.875 (3) Å	θ = 2.4–26.3°
*c* = 10.023 (5) Å	µ = 0.09 mm^−^^1^
β = 117.314 (6)°	*T* = 298 K
*V* = 507.4 (4) Å^3^	Plate, colourless
*Z* = 2	0.55 × 0.41 × 0.09 mm

### Data collection

**Table:**

Bruker SMART CCD area-detector diffractometer	890 independent reflections
Radiation source: fine-focus sealed tube	575 reflections with *I* > 2σ(*I*)
graphite	*R*_int_ = 0.028
φ and ω scans	θ_max_ = 25.0°, θ_min_ = 4.1°
Absorption correction: multi-scan (*SADABS*; Sheldrick, 1996)	*h* = −11→11
*T*_min_ = 0.953, *T*_max_ = 0.992	*k* = −6→6
2508 measured reflections	*l* = −11→11

### Refinement

**Table:**

Refinement on *F*^2^	Primary atom site location: structure-invariant direct methods
Least-squares matrix: full	Secondary atom site location: difference Fourier map
*R*[*F*^2^ > 2σ(*F*^2^)] = 0.041	Hydrogen site location: inferred from neighbouring sites
*wR*(*F*^2^) = 0.123	All H-atom parameters refined
*S* = 1.01	*w* = 1/[σ^2^(*F*_o_^2^) + (0.0655*P*)^2^ + 0.0552*P*] where *P* = (*F*_o_^2^ + 2*F*_c_^2^)/3
890 reflections	(Δ/σ)_max_ = 0.001
97 parameters	Δρ_max_ = 0.16 e Å^−^^3^
0 restraints	Δρ_min_ = −0.15 e Å^−^^3^

### Special details

**Table:**

Geometry. All e.s.d.'s (except the e.s.d. in the dihedral angle between two l.s. planes) are estimated using the full covariance matrix. The cell e.s.d.'s are taken into account individually in the estimation of e.s.d.'s in distances, angles and torsion angles; correlations between e.s.d.'s in cell parameters are only used when they are defined by crystal symmetry. An approximate (isotropic) treatment of cell e.s.d.'s is used for estimating e.s.d.'s involving l.s. planes.
Refinement. Refinement of *F*^2^ against ALL reflections. The weighted *R*-factor *wR* and goodness of fit *S* are based on *F*^2^, conventional *R*-factors *R* are based on *F*, with *F* set to zero for negative *F*^2^. The threshold expression of *F*^2^ > σ(*F*^2^) is used only for calculating *R*-factors(gt) *etc*. and is not relevant to the choice of reflections for refinement. *R*-factors based on *F*^2^ are statistically about twice as large as those based on *F*, and *R*- factors based on ALL data will be even larger.

### Fractional atomic coordinates and isotropic or equivalent isotropic displacement parameters (Å^2^)

**Table:**

	*x*	*y*	*z*	*U*_iso_*/*U*_eq_	
N1	0.7159 (2)	−0.2597 (3)	0.65617 (19)	0.0547 (6)	
N2	0.5861 (2)	−0.3398 (3)	0.66438 (18)	0.0555 (6)	
C1	0.7050 (2)	−0.0820 (4)	0.5729 (2)	0.0466 (6)	
C2	0.56377 (19)	0.0384 (3)	0.49395 (18)	0.0390 (5)	
C3	0.5474 (2)	0.2352 (3)	0.4062 (2)	0.0450 (5)	
C4	0.6864 (3)	0.3211 (5)	0.3949 (3)	0.0581 (7)	
C5	0.8354 (3)	0.2567 (4)	0.5326 (3)	0.0647 (7)	
C6	0.8445 (3)	0.0004 (4)	0.5597 (3)	0.0621 (7)	
H1	0.682 (2)	0.250 (4)	0.305 (3)	0.065 (6)*	
H2	0.678 (2)	0.487 (4)	0.382 (2)	0.076 (7)*	
H3	0.930 (3)	0.308 (4)	0.522 (3)	0.086 (8)*	
H4	0.839 (3)	0.340 (4)	0.623 (3)	0.079 (7)*	
H5	0.846 (2)	−0.086 (4)	0.470 (2)	0.072 (7)*	
H6	0.936 (3)	−0.039 (4)	0.649 (3)	0.081 (7)*	

### Atomic displacement parameters (Å^2^)

**Table:**

	*U*^11^	*U*^22^	*U*^33^	*U*^12^	*U*^13^	*U*^23^
N1	0.0567 (12)	0.0508 (12)	0.0500 (10)	0.0164 (9)	0.0187 (9)	0.0061 (8)
N2	0.0652 (12)	0.0458 (11)	0.0474 (11)	0.0115 (9)	0.0189 (9)	0.0086 (8)
C1	0.0501 (12)	0.0467 (12)	0.0395 (10)	0.0102 (10)	0.0175 (9)	−0.0019 (10)
C2	0.0445 (11)	0.0386 (11)	0.0306 (9)	0.0072 (8)	0.0146 (8)	−0.0029 (8)
C3	0.0574 (13)	0.0386 (12)	0.0351 (10)	0.0051 (10)	0.0178 (9)	−0.0010 (9)
C4	0.0737 (17)	0.0505 (15)	0.0555 (14)	−0.0061 (12)	0.0344 (13)	−0.0027 (12)
C5	0.0595 (15)	0.0680 (17)	0.0681 (16)	−0.0087 (13)	0.0306 (13)	−0.0101 (13)
C6	0.0484 (14)	0.0707 (18)	0.0650 (16)	0.0111 (11)	0.0242 (13)	−0.0033 (12)

### Geometric parameters (Å, °)

**Table:**

N1—C1	1.310 (3)	C4—C5	1.518 (3)
N1—N2	1.381 (2)	C4—H1	0.98 (2)
N2—C3^i^	1.309 (3)	C4—H2	0.98 (2)
C1—C2	1.417 (3)	C5—C6	1.526 (3)
C1—C6	1.499 (3)	C5—H3	1.01 (2)
C2—C2^i^	1.373 (3)	C5—H4	1.01 (2)
C2—C3	1.417 (3)	C6—H5	1.04 (2)
C3—N2^i^	1.309 (3)	C6—H6	0.95 (2)
C3—C4	1.492 (3)		
			
C1—N1—N2	120.01 (17)	C5—C4—H2	111.0 (13)
C3^i^—N2—N1	120.67 (18)	H1—C4—H2	109.4 (18)
N1—C1—C2	121.89 (19)	C4—C5—C6	111.2 (2)
N1—C1—C6	120.07 (18)	C4—C5—H3	111.1 (13)
C2—C1—C6	118.0 (2)	C6—C5—H3	109.2 (14)
C2^i^—C2—C3	118.3 (2)	C4—C5—H4	108.6 (14)
C2^i^—C2—C1	117.8 (2)	C6—C5—H4	110.1 (13)
C3—C2—C1	123.94 (17)	H3—C5—H4	106.5 (19)
N2^i^—C3—C2	121.33 (19)	C1—C6—C5	110.70 (19)
N2^i^—C3—C4	120.3 (2)	C1—C6—H5	107.1 (12)
C2—C3—C4	118.39 (18)	C5—C6—H5	110.5 (12)
C3—C4—C5	111.3 (2)	C1—C6—H6	109.2 (14)
C3—C4—H1	105.8 (12)	C5—C6—H6	111.1 (15)
C5—C4—H1	110.6 (12)	H5—C6—H6	108.2 (19)
C3—C4—H2	108.6 (13)		

Symmetry codes: (i) −*x*+1, −*y*, −*z*+1.
